# Therapeutical Notes

**Published:** 1898-12-03

**Authors:** 


### THERAPEUTICAL NOTES.

### Intestinal Antisepsis.

A useful remedy for auto-intoxication, colic, and intestinal catarrh may be found in the formula of Dr. J. de Maximovitch (*Medical Record*, September 24th, 1898):—

Beta naphthol, gr. 45.

Chloroform, ♏ 15.

Oil of peppermint, ♏ 5.

Castor oil, ℥3.

M. S.—A tablespoonful to be given as a dose.

### Rheumatism and the Neuralgic Pains of Influenza.

Capitan (*Klin. Therap. Wochenschrift*, April, 1898) advises that the following should be applied twice daily:—

℞ Salicylic acid.
Oil of turpentine, āā 6 parts.
Extract of belladonna, 0·20.
Vaseline.
Lanoline, āā 15.

M.—Make an ointment.

### Hyperchlorydria.

The following prescription is given by Boas (*Diagnostik und Ther. der Magenkrankheiten. Gaz. Hebd. de Médecine et de Chir. Mai* 15):—

℞ Sodium sulphate, 30 parts.
Potass. sulphate, 5.
Borax, 10.
Sodium chlorate, 30.
Sodium carbonate, 25.

M. S.—Half a teaspoonful dissolved in a wineglass of warm water, to be taken thrice daily, viz., before breakfast and two hours before luncheon and dinner.

### Gastric Ulcer.

Dreschfeld (*British Medical Journal*, March 12th, 1898) pointed out the dangers of the stomach tube in gastric ulcer, but had obtained great success by giving 30, 40, or even 50 grains of bismuth subnitrate three times a day, suspended in water where ordinary doses failed. In acid dyspepsia also it rapidly relieved the symptoms.

